# COVID-19: Adaptations and changes at Guinness Eye Centre, Nigeria

**Published:** 2020-09-01

**Authors:** Adeola Onakoya

**Affiliations:** 1 Professor of Ophthalmology and Head: Department of Ophthalmology, Lagos University Teaching Hospital/College of Medicine University of Lagos, Nigeria.

**The COVID-19 pandemic has brought unprecedented challenges for eye care in Lagos, Nigeria.**

**Figure:**
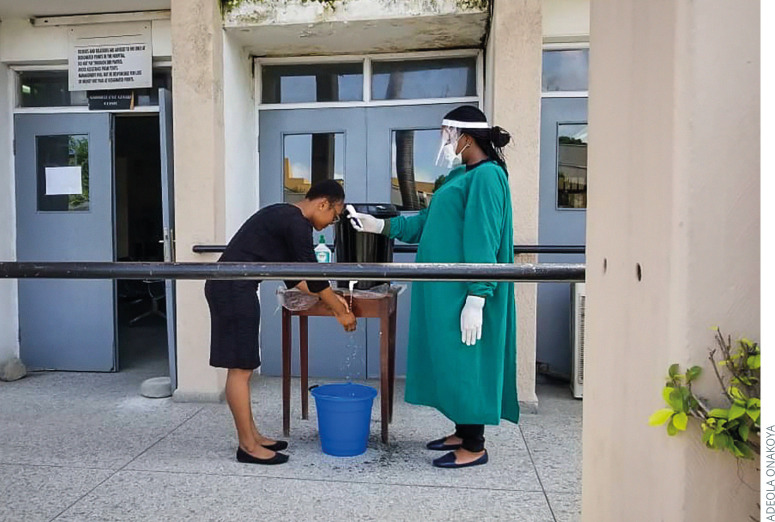
A nurse checks the temperature of patients before they enter the eye centre. **NIGERIA**

Guinness Eye Centre (GEC) is the Eye Department of Lagos University Teaching Hospital and College of Medicine, University of Lagos, Nigeria; it offers specialist eye care to between 13,000 and 15,000 people per year from Lagos, neighbouring states, and other countries.

The first person to test positive for COVID-19 in Nigeria was identified in Lagos on 27 February 2020. With Lagos becoming the epicentre of COVID-19 in the country, it was vital to adapt eye services to prevent further spread. From 28 February, GEC added more infection control measures whilst continuing to offer all its usual services.

- An auxiliary nurse carried out triage at the entrance to GEC, using an infrared thermometer to check patients’ temperature. Patients with a high temperature and/or any history of cough or sore throat were treated as suspected COVID-19 cases (Figure 1).
  **Figure 1:**
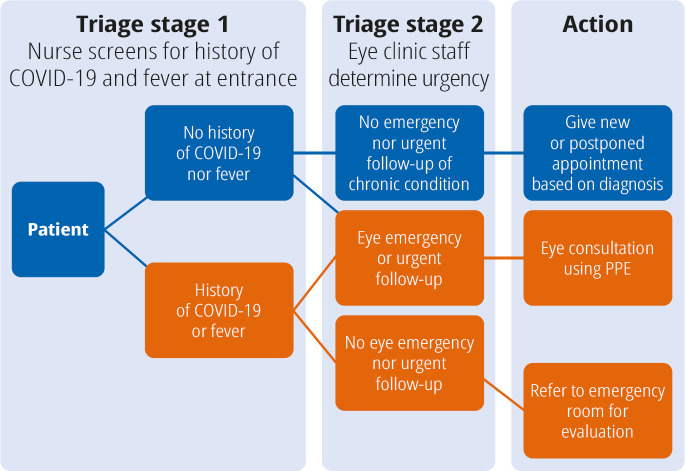
Protocol for patient triage during the COVID-19 pandemic
- Hand sanitisers, water, and soap were available at all entrances so people could wash their hands before entering.
- Alcohol disinfectants were used to clean surfaces and equipment in consulting rooms and in clinical and administrative areas. Slit lamps were disinfected after each patient.
- All doctors and nurses involved in patient care received face masks, eye protection (goggles) and gloves.

A month later, there was a sudden surge in the number of confirmed COVID-19 cases in Lagos, with suspected community transmission. Hospital managers decided to reduce services and take further measures to protect staff members and patients.

- In order to control the flow of people entering and leaving the hospital, we sealed the back entrance so only the main entrance was kept open.
- Attending physicians and nurses used face shields and protective wear, for example, using adapted theatre gowns as personal protective equipment (PPE) when attending patients.
- Intraocular pressure assessment was performed with non-contact tonometer, and only when this was essential.
- Staff members encouraged and supported to practice social distancing, i.e., stay 1-2 metres away from each other.
- Patients requiring non-urgent follow-up were given extended appointments (i.e., they could come back at a future date).
- Elective surgery was postponed until further notice
- Clinic consultations were restricted to patients with urgent and emergent conditions only, including recent surgery, retinoblastoma, retinopathy of prematurity, unexplained red eye, trauma, and sudden vision loss. Staff members shared contact numbers with patients and, if needed, contacted them to provide further information.
- Staff members were put on a rota and attended only once or twice per week.
- We started to offer some of our training and teaching activities by webinar, which has been very well received.

**Figure:**
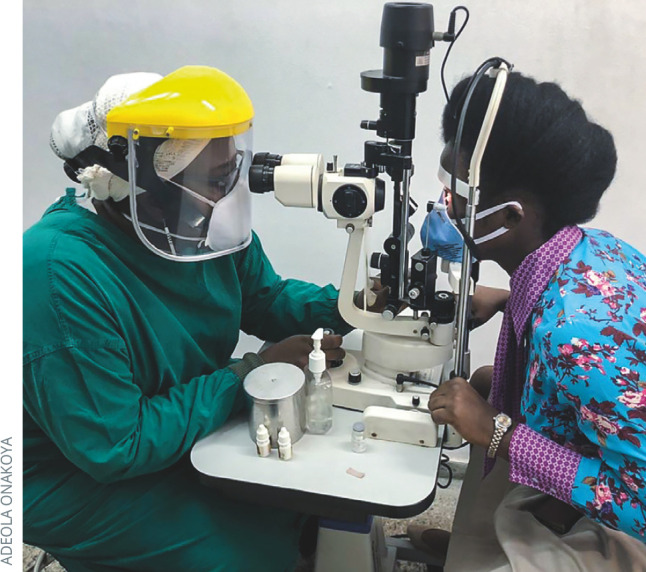
Physicians and nurses wore adapted theatre gowns and used face shields and face masks when attending to patients. **NIGERIA**

Going forward at GEC after lockdown involves a gradual return to full services, starting with around 30% of the normal consultations and offering staggered appointment times. Stringent measures are being introduced to reduce the number of people in the waiting area, e.g., by allowing patients to be accompanied by no more than one supporting family member, if needed. Everyone in the clinic will be expected to practice social distancing, wash and disinfect their hands, and wear face masks. Appointments for patients with stable chronic conditions such as glaucoma or refractive errors will be postponed.

Services will be evaluated and reviewed every two weeks. Elective surgery is still on hold until a return to full activities, which depend on a significant decline in the COVID-19 infection rate and a return to normal activities in the country.

## Acknowledgements

*With thanks to the resident doctors at Guinness Eye Centre, Lagos University Teaching Hospital for their help with the images in this article.*

**Useful resources**

1. Nigeria Centre for Disease Control (NCDC) COVID-19 dashboard. **http://COVID19.ncdc.gov.ng**
2. American Academy of Ophthalmology Advisory on COVID-19. **http://aao.org/coronavirus**

